# The significance of advanced COVID-19 diagnostic testing in pandemic control measures

**DOI:** 10.7150/ijbs.72837

**Published:** 2022-07-11

**Authors:** Hang Fai Kwok

**Affiliations:** 1 Institute of Translational Medicine, Faculty of Health Sciences, University of Macau, Avenida de Universidade, Taipa, Macau SAR; 2 Department of Biomedical Sciences, Faculty of Health Sciences, University of Macau, Avenida de Universidade, Taipa, Macau SAR

**Keywords:** SARS-CoV-2, Diagnostic testing, Pandemic conditions, Viral Variants

## Abstract

During the 2 years since the start of the novel coronavirus disease 2019 (COVID-19) pandemic, the scientific world made an enormous effort to fight against this disease caused by severe acute respiratory syndrome coronavirus 2 (SARS-CoV-2), which has high transmissibility. Advancements in vaccine and treatment strategies have reduced both the hospitalization and mortality rates. However, the virus has shown its ability to evolve and evade from our COVID-19 combating armamentaria by the most common evolution mechanism—mutation. Diagnostic testing has been the first line of defense following the identification of the causative agent. Ever since, the scientific community has developed nuclei acid-based, antigen-based, and antibody-based diagnostic tests, and these testing methodologies are still playing a central role in slowing down viral transmission. These testing methods have different sensitivity and specificity and could be optimally used in areas facing different challenges owing to different level and conditions of COVID-19 outbreak. In this review, we discuss these testing methodologies as well as the considerations on how to apply these diagnostic tests optimally in the community to cope with the ever-changing pandemic conditions.

## Introduction

As of February 2022, it has been almost 2 years since the coronavirus disease 2019 (COVID-19) pandemic started, and the pandemic has claimed more than 5 million lives from almost 4 billion infections 1. Before the vaccines targeting severe acute respiratory syndrome coronavirus 2 (SARS-CoV-2) were developed and effective therapeutic agents that could decrease the rates of severe disease were administered, hospitalization and death rates were very high, and most of the countries were combating COVID-19 by physical measures such as mask mandates and social distancing to slow the spread of the virus. Further, making diagnostic tests readily available to identify COVID-19 infections in combination with contact tracing and quarantine guideline helped to fight against the COVID-19 outbreak by slowing down SARS-CoV-2 transmission.

In these 2 years, considerable progress has been made with respect to development of vaccines against SARS-CoV-2 and treatment for COVID-19. Indeed, several vaccines have already been approved by China National Medical Products Administration, United States Food and Drug Administration, and the World Health Organization (WHO) for emergency use to prevent disease transmission or severe disease 2-4, while several drug molecules or antibody biologics have also been approved for emergency use for COVID-19 management in some countries 5. These innovations have been rapidly put into pandemic control policies, strategies, and actions, and have become an important aspect of controlling the pandemic in many countries 6. Although multiple phase III clinical trials have shown highly positive results on the effectiveness and safety of several vaccines 7-10 and therapeutic agents 11-15, vaccines and therapeutic agents are far from perfect with respect to preventing transmission or curing patients who developed severe disease. In particular, their efficacy may vary with the emergence of new SARS-CoV-2 variants. To date, 12 important variants have been identified as important by the WHO, and few of these have been grouped as variants of concern—such as Alpha (B.1.1.7), Beta (B.1.351), Delta (B.1.617.2), and Omicron (B.1.1.529)—owing to their high level of transmissibility resulting in several waves of outbreak in various countries 16. In many of these new COVID-19 outbreak waves, the newly emerged variants replaced the previous variant and became the dominant one. This is actually a limitation of the above-mentioned phase III randomized clinical trials studying vaccines and therapeutic agents, where the original dominant variant at the beginning of the trial may have slowly faded out. At the time when the clinical trials end and the data become analyzable, a newly emerged variant has already become the dominant and established variant. This variant displacement can happen in a very short period of time, and we have seen how Delta and Omicron evolved and became the dominant variant in several countries 17. This displacement, however, will lead to uncertainties on the efficacy of vaccines and drugs, which have been shown in clinical trials as being highly effective against the original dominant variant but may have no or limited efficacy data available for the newly emerged variant. Studies have already demonstrated that the effectiveness of a vaccine could be different against different variants, thereby resulting in different level of vaccine breakthrough 18-21 and even different level of protection against severe disease and hospitalization towards new variants 22.

Although various vaccines have been approved for emergency use, the third and fourth waves of the pandemic because of the novel delta and omicron variants have confirmed that there could be a significant number of vaccine breakthrough cases when newly emerged variants take over as the dominant variant during the COVID-19 outbreak. Other than vaccination and treatment, another effective way to manage and control the COVID-19 outbreak, used throughout the pandemic, is diagnostic testing. Diagnostic tests can be readily modified once the genome sequence of the new variant has been fully revealed. Diagnostic testing has always played an important role in the control of infectious disease; indeed, lack of access to high-level diagnostics has been a major contributor to the existing health burden 23. Indeed, lack of reliable diagnostic tests was a major concern during the early phase of the COVID-19 pandemic, and inventing a diagnostic test with acceptable sensitivity (a measure on the ability of a test to identify true positive case) and specificity (the ability of a test to identify true negative case) was also a major focus of early scientific research for development of COVID-19 controlling strategy. Improving these parameters will therefore improve the accuracy of the diagnostic test. Since the beginning of the pandemic, enormous efforts have been made to develop and improve diagnostic methods, which is essential for accurate case identification 24, because identification of infected individuals in a timely and systematic manner has been shown to play a critical part in controlling the COVID-19 pandemic 25. Diagnostic tests when used together with appropriate guidelines in contact tracing, quarantine, and isolation can slow disease transmission; when coupled with advance forecasting and modeling, testing can also improve intervention planning and disease controlling strategies. Ultimately, diagnostic testing plays an essential role in the management of the pandemic, and a proper policy and guidelines on how to use different diagnostic tests could greatly improve the outcome of the pandemic on patients and healthcare, social, and economic perspectives.

Given the speed with which new variants of SARS-CoV-2 have emerged throughout the pandemic, the increasing transmissibility of the newly emerged variant, and the uncertainties in vaccine breakthrough and treatment efficacy for the newly emerged variants, making diagnostic tests available; reducing the turnaround time; and increasing the sensitivity, specificity, and reducing costs should be paramount in the fight against the pandemic. We believe that diagnosis will remain one of the most important armamentaria even in the late stage of this COVID-19 pandemic. In this review, our goal is to discuss different methodologies of COVID-19 diagnostic tests, the strengths and limitations of these different diagnostic tests, and how these tests help different communities in combating against the COVID-19 outbreak.

## The methodologies of COVID-19 diagnostic tests

In the very early phase of this pandemic, no diagnostic test was available for patients who become very ill in the initial outbreak of viral pneumonia, which was thought to be caused by an unknown microbial agent. Diagnosis is mainly based on the patient's exposure history as well as clinical manifestations. Subsequent research identified the novel coronavirus as the causative agent, and the complete genome sequence of this novel coronavirus, later named as SARS-CoV-2, was obtained from samples from patients in Wuhan by next generation sequencing 26. With the availability of the SARS-CoV-2 genome sequence, the protocol for molecular diagnoses of COVID-19 based on real-time RT-PCR was developed and published by the WHO and this protocol became the basis of commercial diagnostic kit development 27. However, most of the early testing protocols had limitations including sensitivity, specificity, and background cross-reactivity. Moreover, diagnostic tests were required to be processed at off-site centralized laboratories, requiring a long turnaround time with almost a week needed for results to become available 27-29. Some of these limitations were later improved by newly developed commercial assays that have their limit of detection-analytical sensitivity better defined than previous tests. Some of the commercially available real-time RT-PCR diagnostic tests for COVID-19 have a sensitivity of around 95% and a detection limit of as low as 10 copies/reaction, allowing accurate and early detection of infection even when the viral load is relatively low at the initial infection phase 30. In addition, to address the long turnaround time, a rapid real-time molecular assay has also been developed, where a positive result can be produced within 5 min and a negative result, within 13 min 27. For most of the molecular diagnostic test, specimens including nasopharyngeal swabs or aspiration, oropharyngeal swab, sputum, and even saliva, which are relatively easily collected from suspected infected patients, have been validated as appropriate for RNA extraction 31, 32 while specific target regions including ORF1ab, RdRp, N, and S genes are most commonly used by commercial platforms to detect viral infection by real-time RT-PCR 33. Molecular diagnosis using conventional real-time RT-PCR has remained the gold standard throughout the pandemic to ensure test results to be accurate and sensitive enough, which then allow the subsequent quarantine arrangement and contact-tracing procedures. However, most of the real-time RT PCR diagnostic tests are still time consuming, laborious, and require specialized equipment and trained personnel to ensure reliable test results 34. Until the omicron strain emerged and took over as the most dominant SARS-CoV-2 variant across the globe with infection that leads to milder symptoms but a much faster transmission 35, the limitations of real-time RT-PCR, especially on its turnaround time, led us to re-evaluate what could be the optimal diagnostic method for this newly emerged variant.

Antibody-based serology testing has once been used as a tool to identify COVID-19-recovered patients to understand the number of infected patients versus the number of recovered patients for better estimation of the outbreak trends 36. Although some rapid antibody tests have been developed that are easy to use, such as finger-prick for whole blood, and have a relatively short turnaround time of 15-20 min 33, there are few disadvantages of antibody-based serology testing that make it less suitable for diagnostic use during the viral outbreak. These disadvantages include the requirement of using blood instead of respiratory tract samples, because antibodies are only present in the blood, and lack the capacity to identify patients early in the infection phase, because the antibody level may not be at detectable levels in the early phase of the infection. The long sustaining antibody level post infection also results in the inability to distinguish patients from acute infection to recovery phase, as a high antibody level may persist for a prolonged period since infection. Indeed, antibody tests can have an accuracy as low as 30% in the first week after symptom onset which increases in the 2^nd^ and 3^rd^ weeks to 70% and >90% accuracy, respectively 37. Hence, the WHO has issued guidance restricting the use of antibody-based serology test to epidemiological studies only instead of for diagnostic purpose 36.

Another type of test that has increasing popularity at the later stage of the pandemic is antigen-based diagnostic tests. The mechanism of antigen-based testing method is to detect viral surface proteins that are produced during the active phase of SARS-CoV-2 infection 33 without the need for expensive equipment or highly skilled and trained personnel. Rapid antigen detection test was developed to provide timely result of COVID-19 infection using samples collected from nasopharyngeal or oropharyngeal swabs from patients. The accuracy of the antigen test can be influenced by factors including the time of sample collection relative to the infection, the quality of the sample and the reagents, and the viral load in the collected specimens 38. The sensitivity in general is lower than real-time RT-PCR 38 but more comparable with real-time RT-PCR in the acute phase of infection when the viral load and infectivity is the highest. Although a negative result from antigen-based testing cannot always exclude a SARS-CoV-2 infection, it could be very useful in a high prevalence setting during an active outbreak of COVID-19 as the test is easy to administer with a relatively reliable sensitivity and specificity and is possible to run at a much higher volume when available compared to real-time RT-PCR 33, 39.

## Considerations of optimal COVID-19 diagnostic tests for SARS-CoV-2 outbreak

**Figure 1:**
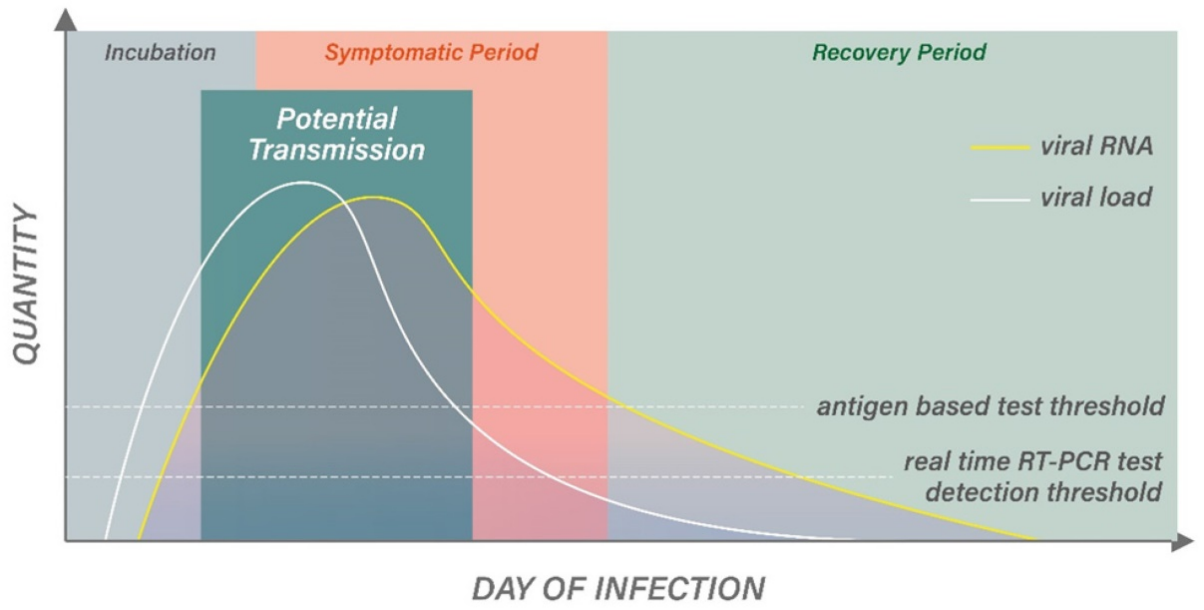
An illustration of the changes in viral load or RNA levels during different infection phases in relation to the detection limits on real-time RT-PCR and antigen-based diagnostic tests

Despite the availability of different diagnostic techniques and commercial kits, an accurate diagnosis of COVID-19 infection requires the proper use of the tests based on the type of samples and the timing of sample collection relative to the potential infection, such as the moment of the patients' exposure and suspected infection, as well as their medical history and clinical manifestations 40, 41. For example, molecular-based real-time RT-PCR is more sensitive and may be more suitable at the very initial phase of infection to detect a relatively low level of RNA copies when a patient transitions from an incubation phase to an active infection phase. Therefore, even before the onset of the symptoms, real-time RT-PCR may still be able to detect the viral infection. During the active infection phase with symptoms onset, both real-time RT-PCR and antigen-based tests may be sensitive enough to yield a positive result owing to the high viral load, but the antibody level may still remain at an undetectable level for antibody-based tests. The sensitivity of the antigen-based test at first was significantly lower than the real-time RT-PCR detection method but with a comparable specificity. Although antigen-based diagnostic methodology plays a limited role in detecting other viral infection because of its lower sensitivity than real-time RT-PCR, it is particularly useful for detecting SARS-CoV-2 infection as the viral load is high in the nasopharynx of infected patients within the first week of disease, allowing antigen-based testing to identify patients early when they become infectious (Figure 1) 42. As such, later in SARS-CoV-2 pandemic, the antigen-based testing method showed improved sensitivity to an average of around 94% and become comparable to the real-time RT-PCR test during the first week of infection, when the viral load was usually highest during the infection. The sensitivity of the antigen-based test was 98% at a viral load corresponding to a Ct value ≤25 by real-time RT-PCR and was usually negative when the Ct value was >30 38, allowing antigen-based diagnostic tests to give relatively reliable results during the active infection phase.

Upon entering the later phase of infection where the virus is no longer transmissible owing to a lower viral load, the more sensitive real-time RT-PCR test may still give a positive result that could lead to unnecessary quarantining or isolation (Figure 1). Because real time RT-PCR tests are usually extremely sensitive, which can detect RNA at a copy number between 20 and 100 copies/mL, the positivity may last for days beyond the period when the disease is no longer transmissible, which requires an RNA concentration of >100 copies/mL 42. Although real-time RT-PCR is a semi-quantitative technique that potentially a threshold can be established based on the cycle number (Ct value), it is still inversely correlated with the viral load, and hence transmissibility. Candel et al. and Cliotti et al. listed a few limitations regarding the establishment of a threshold for real-time RT-PCR for infectivity. Especially, how the Ct value can be influenced by various factors including quality of the sample, delay in sample processing, incorrect specimen handling, problem during shipment, and variability in different commercial platforms and systems 33, 42. These limitations also potentially led to false negative results. Continuous efforts have been made to predict the viral load based on laboratory Ct value, but further optimization is required to bring the research to routine use in a clinical setting 43. Due to these limitations, a complementary or alternative testing method, antigen detection tests, based on lateral immunochromatography has been developed. Conversely, an antigen-based test may give a result that could be more indicative of infectivity of the patients. After the infection phase and when the symptoms resolve, antibody test may be the only diagnostic method that can identify patients who have survived COVID-19 and fully recovered from the SARS-CoV-2 infection, as both real-time RT-PCR and antigen based test would show negative results 44.

The considerations of which testing method should be used can be very different at a population level versus an individual level. As each diagnostic test has different sensitivity and specificity in different phases of viral infection, using a specific type of diagnostic test can lead to different goals. For example, real-time RT-PCR is to identify infected individuals early in the infection cycle due to the high sensitivity of this methodology, which can ultimately stop viral transmission earlier and more effective. In addition, an antigen-based diagnostic test is relatively easy to use where patients can do multiple tests at home to monitor the development of disease after exposure to an infected individual. In contrast, an antibody-based test can be used to identify individuals who have been recovered from infection informing public health experts of the outbreak's severity.

During the second wave of the pandemic with the new variants adopting a higher transmissibility, the diagnostic demand significantly increased. As real-time PCR diagnostic testing requires specific equipment and trained personnel, it is difficult to meet the increasing demand in such a short time. The inability to meet demands as the volume of test requested increases will result in delays in issuing results, which then delay the decisions on isolating infected COVID-19 patients. However, with the diagnostic test reaches its capacity in a given geographic area, patients with less medical complications or those who have close contact with COVID-19 patients may not be able to access diagnostic test, resulting in delay in decision of quarantine too. Based on these limitations of real-time PT-PCR diagnostic technology, it cannot be used as a sole diagnostic technology for a country to fight against the pandemic.

**Figure 2:**
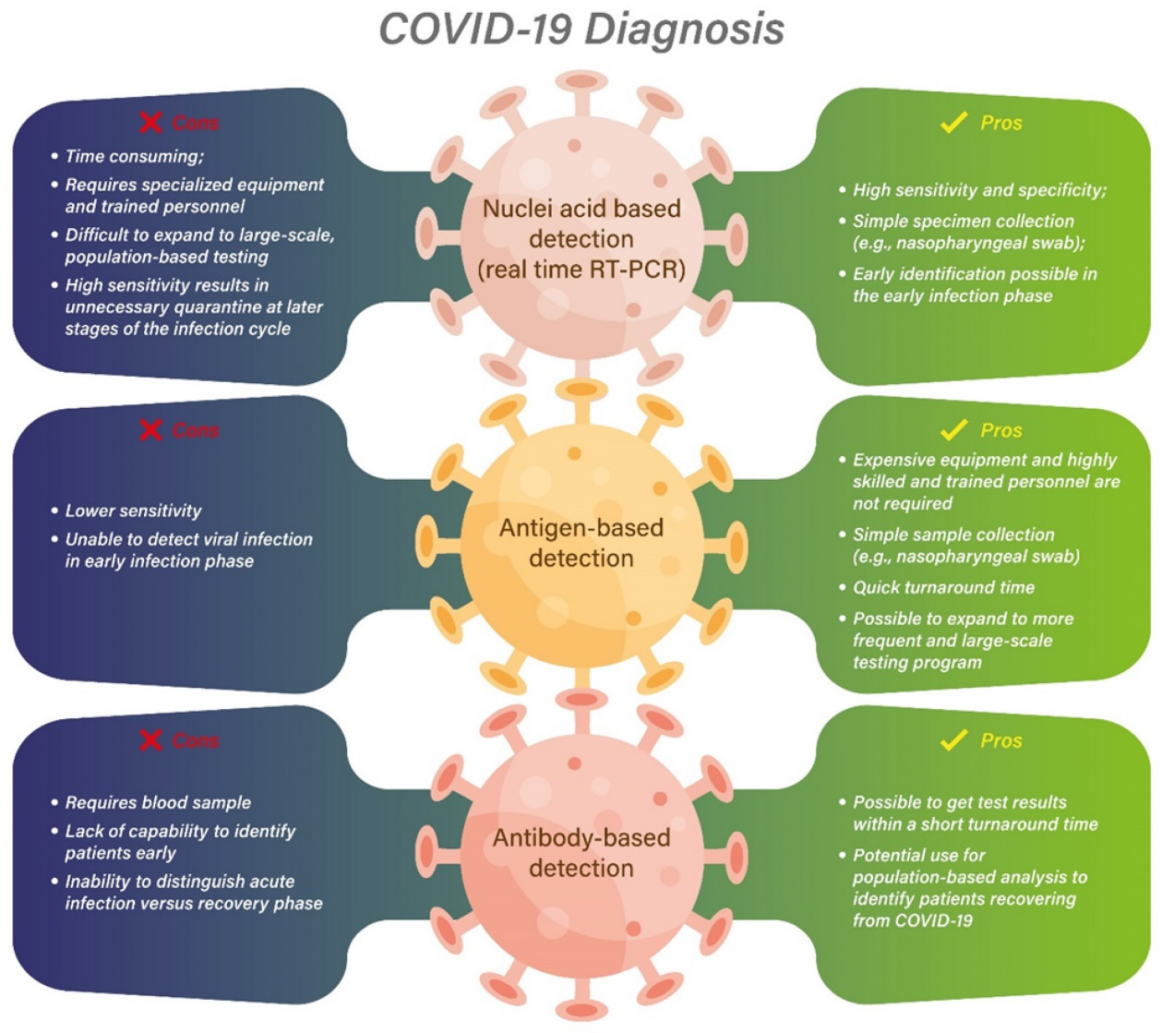
The pros and cons of different diagnostic testing methodologies

Although the later development of the rapid antigen-based diagnostic test results in a higher sensitivity and specificity than its original version, the false positive and negative rate would still be impacted by the prevalence of COVID-19 within the community, and the community setting including the population density, quarantine guidelines, and the vaccination rate. In a community with high prevalence of COVID-19, a positive antigen-based test may be enough, but a negative test may need to be re-confirmed with an additional test. On the other hand, with a low prevalence and no exposure history, a negative antigen test may be sufficient to rule out infection 42. Therefore, an effective diagnostic test, from a population perspective, should have the ability to identify most infected patients, regardless of the phase of infection and whether they are symptomatic, but when they are still able to transmit their infections to others. Identifying such patients can help to stop the pandemic by isolating these patients 45. However, an effective diagnostic test should over-diagnose positivity as that could result in isolating COVID-19 patients who are no longer infectious to the community. This unnecessary quarantine and isolation should be avoided. In summary, different testing methodologies have its unique advantages and disadvantages relative to the different individual infection conditions and population-based outbreak situations (Figure 2).

Different algorithms have been proposed for testing with different conditions, and real-time PCR and antigen-based tests can be complimentary to each other in different scenarios to improve testing accuracy and speed 42. Nonetheless, the one universal aspect is that for diagnostic testing that could effectively stop the spread of a virus with high transmissibility like SARS-CoV-2, a huge daily testing volume is required as suggested by a network-based model created by China research group 46. During an outbreak with dramatic increases in case numbers, the improvement in the testing capabilities and capacity can be the most important objective to achieve to cope with the testing demand 47. Indeed, repeat and regular testing has been shown to be effective in identifying, isolating, and preventing infected patients including asymptomatic spreaders 45. In addition to multiple tests with different methodologies that would complement each other to increase sensitivity, multiple tests with the same methodology may be required to identify infected patients; a mathematical model has been developed which suggests an almost 100% sensitivity is required for a diagnostic test to contain the spread of this variant, and hence a triplet test system may be needed to minimize the spread of such variants 48. In addition to early identification of infections, diagnostic tests can also be used in combination with other safety precautions to prevent viral spread. A recently completed randomized controlled open-label trial assessing the effectiveness of same-day screening of attendees with antigen-based rapid testing in combination with masking and increased air ventilation has shown that these interventions can be a safe approach for indoor mass gathering events during the COVID-19 outbreak 49. To implement all the above testing measures to better fight against SARS-CoV-2, the capability to increase testing capacity has become the major focus across many countries worldwide.

Increased testing rate through mass or universal testing has been suggested to be an effective strategy to slow down the spread of viral infection. Although effective disease detection strategy is an integral part of pandemic control policy to minimize the numbers of infections and death during the COVID-19 pandemic 50, not all countries are ready to implement different testing strategies during various level of outbreak situations, especially when it comes to a severe outbreak where capacity of mass testing and case isolation may be required 51. During the early phase of COVID-19 outbreak, the pandemic control policy focused on "early identification, early isolation and early treatment" has succeeded in controlling the spread of the virus, especially in some high population density cities/regions such as Hong Kong and Macau. However, due to the rapid evolution of SARS-CoV-2, the policy of "early identification, early isolation and early treatment" has to work along with increasing the vaccination rate at the same time to decrease the speed of viral spread. In some countries such as the United Kingdom, increased testing capacity allowed for mass asymptomatic testing at a frequency of twice per week for everyone in England. Although this approach was able to identify infected and asymptomatic patients and seemed to prevent transmission in certain cities such as Liverpool, the participation rate was low and negative attitudes were received from the public due to concerns on the accuracy of the tests as well as the effectiveness of the biweekly testing policy 52. In the Netherlands, a national testing policy of weekly testing of all individuals in nursing homes during the SARS-CoV-2 outbreaks was recommended, but could only be partially implemented because of people's reluctance for undergoing serial testing without symptoms 53. The hesitancy of participation in mass testing is not a Europe-specific observation; a cross-sectional study conducted in Hong Kong showed that a majority of survey interviewees were unwilling to participate in a universal community testing program, hence limiting the effectiveness of implementing such testing policies during a severe SARS-CoV-2 outbreak 54.

## Conclusions

Challenges to control COVID-19 transmission still remain as not all countries have adequate testing resources and the capacity to implement mass or even universal testing. There are also concerns on the variability of test performance among different diagnostic methodologies impacting the accuracy of the test as described above, and the uncertainties in testing sensitivity towards newly emerged variants 55. The hesitancy of the public to engage in testing is another challenge that governments are facing when implementing testing policies. Indeed, different countries are facing different situations as they have different epidemiology and testing requirements to fight against the pandemic. Molecular testing using real-time RT-PCR and antigen-based diagnostic tests have complimentary roles in pandemic management, and the balance of their benefits and risks are important considerations in testing policy and its implementation 55. As the COVID-19 pandemic continues to evolve and new variants emerge, the scientific world has to be prepared to optimize and innovate the diagnostic methodology and capacity to cope with the ever-changing face of the pandemic.
